# Regulatory mechanisms of the gut microbiota-short chain fatty acids signaling axis in slow transit constipation and progress in multi-target interventions

**DOI:** 10.3389/fmicb.2025.1689597

**Published:** 2025-11-24

**Authors:** Shi Hong Liu, Xue Fang Yang, Lei Liang, Bin Bin Song, Xue Mei Song, Yong Jun Yang, Mohammed Abdelfatah Alhoot

**Affiliations:** 1 Department of Integrated Traditional Chinese and Western Medicine of Colorectal and Anal Diseases, Affiliated Hospital of North Sichuan Medical College, Nanchong, Sichuan, China; 2 School of Graduate Studies, Postgraduate Centre, Management and Science University, Shah Alam, Selangor, Malaysia; 3 Department of Graduate School, Tianjin University of Traditional Chinese Medicine, Tianjin, China; 4 Department of Traditional Chinese Medicine, Affiliated Hospital of North Sichuan Medical College, Nanchong, China; 5 Department of Surgical Center, Affiliated Hospital of North Sichuan Medical College, Nanchong, China; 6 International Medical School, Management and Science University, Shah Alam, Selangor, Malaysia

**Keywords:** slow-transit constipation, gut microbiota, short-chain fatty acids, FFAR, microbiota–neuro–immune axis, precision nutrition, fecal microbiota transplantation, multi-target interventions

## Abstract

Slow-transit constipation (STC) is an increasingly prevalent disorder that imposes a substantial health and economic burden. Mounting evidence highlights the “gut microbiota–short-chain fatty acid (SCFA)–motility” axis as a central pathophysiological link between dysbiosis and impaired colonic transit. This review synthesizes current knowledge of how SCFAs, especially acetate, propionate and butyrate, shape motility through serotonergic signaling, enteric nervous system modulation, epithelial barrier integrity and immune regulation. Particular attention is devoted to the biased-signaling properties of the SCFA receptors FFAR2 and FFAR3 (free fatty acid receptors 2 and 3, respectively), including emerging data on their heterodimerization. The article then appraises recent randomized controlled trials and meta-analyses of multi-target interventions (dietary fibers, synbiotics, postbiotics, fecal microbiota transplantation, phytochemicals, and small-molecule FFAR agonists) highlighting their efficacy, safety, and translational hurdles. Finally, the authors propose a precision-medicine framework that integrates multi-omics microbiome profiling, metabolomics, and host genetics to enable phenotype-stratified therapy. Key research gaps include limited long-term safety data, heterogeneous human cohorts and the need for large multicenter trials and machine-learning-guided responder prediction. Collectively, the review provides a roadmap for shifting STC management from symptom control to mechanism-based, personalized care.

## 1 Introduction

### 1.1 Epidemiological burden and public health significance of slow transit constipation

Slow transit constipation (STC) represents a major subtype of chronic constipation, affecting approximately 2–27% of the population globally, with significant geographical and demographic variations (Vlismas et al., 2024). The condition disproportionately affects women and the elderly, with prevalence rising to 12.5–30% in individuals over the age of 65 (Daniali et al., 2020). STC imposes a considerable economic burden, with annual healthcare costs exceeding $6.9 billion in the United States alone, and average annual medical expenses per patient surpassing $7,500 (Portalatin and Winstead, 2012). Beyond direct medical costs, STC significantly impairs quality of life, to that of other chronic diseases such as inflammatory bowel disease and diabetes (Ihana-Sugiyama et al., 2016). Recent epidemiological studies have revealed troubling associations between STC and cardiovascular disease, with a Mendelian randomization study demonstrating that hypertension causally increases constipation risk (OR = 3.459, 95% CI: 1.820–6.573), though not the reverse (Wang et al., 2024).

Collectively, these findings underscore the considerable burden STC imposes on both individual health and public healthcare systems. In addition to its high prevalence and demographic disparities, STC contributes to substantial healthcare costs and considerable reductions in quality of life. More critically, emerging evidence suggests that STC may not be merely a localized gastrointestinal disorder but a manifestation of broader systemic dysfunction, as indicated by its potential causal link with hypertension. This underscores the urgency of adopting integrated, multi-system strategies for the diagnosis and management of STC.

### 1.2 The concept of “microbiota-SCFA-gut motility” and its theoretical value

**Figure 1:**
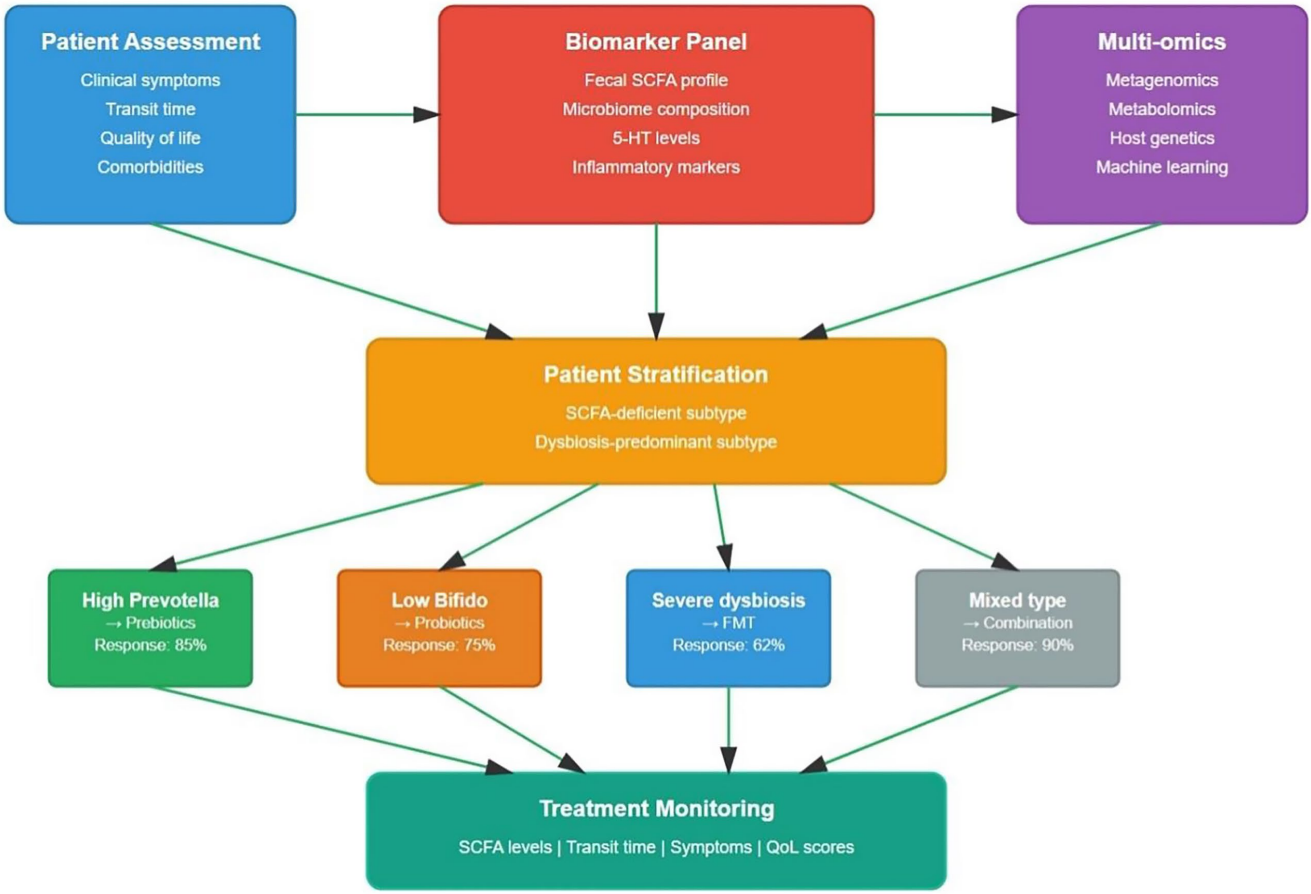
The microbiota-SCFA-gut motility axis in slow transit constipation. Dysbiotic microbiota with a reduced abundance of SCFA-producing bacteria leads to decreased production of short-chain fatty acids (acetate, butyrate and propionate). This reduction impairs FFAR2/3 receptor signaling, diminishes 5-hydroxytryptamine (5-HT) synthesis, decreases the density of interstitial cells of Cajal (ICC) and compromises enteric nervous system (ENS) function. The resulting cascade ultimately reduces smooth muscle contractility, culminating in slow-transit constipation. Red dashed arrows denote inhibitory or reduced effects.

The paradigm of the “microbiota-SCFA-gut motility” axis has emerged as a fundamental framework for understanding STC pathophysiology. Short-chain fatty acids (SCFAs), primarily acetate, propionate, and butyrate, are produced by gut microbiota through fermentation of dietary fibers and represent the major carbon flux from diet through the microbiome to the host (Morrison and Preston, 2016). These metabolites serve as critical mediators linking gut microbiota composition to intestinal motility through multiple mechanisms including direct effects on enteric neurones, modulation of serotonin signaling, and regulation of interstitial cells of Cajal (ICC) function (He et al., 2020). The theoretical importance of this axis is underscored by clinical observations showing reduced fecal SCFA levels, particularly butyrate and propionate, in STC patients, with these reductions correlating directly with constipation severity (Chen et al., 2024). Recent mechanistic studies have demonstrated that microbiota-derived butyrate can rescue STC symptoms by promoting 5-hydroxytryptamine (5-HT) synthesis through upregulation of tryptophan hydroxylase-1 (TPH1), establishing a clear molecular pathway from microbial metabolism to gut motility (Makizaki et al., 2021). In summary, the microbiota–SCFA–gut motility axis in STC is shown in Figure 1.

### 1.3 Review scope, innovation points, and objectives: integrating molecular mechanisms and multidimensional interventions to establish foundation for precision treatment of STC

This review comprehensively examines the regulatory mechanisms of the gut microbiota–SCFA signaling axis in STC and evaluates recent advances in multi-target therapeutic interventions. The novelty of this synthesis lies in integrating molecular, cellular, and clinical perspectives to provide a unified framework for understanding STC pathophysiology. Specifically, we systematically analyze: (1) the molecular mechanisms linking SCFA deficiency to impaired gut motility; (2) the bidirectional relationship between microbiota dysbiosis and SCFA production; (3) emerging multi-target interventions including dietary modifications, probiotics, traditional medicines, and novel receptor-targeted therapies; and (4) the clinical translation potential for precision medicine approaches (Wang et al., 2022).

Unlike previous reviews that focused on single dimensions such as gut motility or microbiota imbalance, this study constructs an integrated “microbiota–SCFA–neuro–immune” interaction network and positions multi-target interventions within a precision medicine framework. By consolidating evidence from over 80 recent studies, the review aims to establish both a theoretical and practical foundation for developing personalized, microbiota-targeted therapies that address the heterogeneous nature of STC and overcome the limitations of current symptom-based treatments (Khalif et al., 2005). In addition, we draw selective insights from peripheral microbiomes: for example, the early-to-mature transition of biofilms on removable dental prostheses parallels shifts in microbial diversity and inflammatory markers. This finding signals how oropharyngeal ecology can index systemic inflammatory “load.” Similarly, exploratory work on topical probiotics and skin-barrier homeostasis highlights cross-site microbiome contributions to whole-body inflammatory and neuroimmune tone (Yacob et al., 2024; Habeebuddin et al., 2022).

## 2 Materials and methods

### 2.1 Literature search strategy and evidence grading

A comprehensive literature search was performed in PubMed, Web of Science, and Scopus up to 11 July 2025. Keywords included “slow-transit constipation,” “gut microbiota,” “short-chain fatty acids,” “FFAR2,” “FFAR3,” “fecal microbiota transplantation,” and related MeSH (Medical Subject Headings) terms. Only articles published in English were considered. The strength of evidence was graded according to the Oxford Centre for Evidence-Based Medicine 2011 criteria (Levels 1a–5). Discrepancies in study selection were resolved by consensus among authors.

## 3 Results

### 3.1 Pathophysiological overview of STC

#### 3.1.1 Colonic transit physiology and enteric neuro-muscular coordination

Normal colonic transit depends on the intricate coordination between the enteric nervous system (ENS), interstitial cells of Cajal (ICC), smooth muscle cells, and the autonomic nervous system. The ENS, often termed the “second brain,” contains approximately 400–600 million neurones organized into myenteric and submucosal plexuses that regulate motility through complex neural circuits (Hao and Young, 2009). ICC serve as intestinal pacemakers, generating slow waves that coordinate smooth muscle contractions, with their density and function being critical determinants of colonic transit time (Friedmacher and Rolle, 2023). In STC, marked reductions in ICC density have been documented, with immunohistochemical studies showing 50–70% decreases in c-Kit-positive cells compared to healthy controls (Lee et al., 2005). Although the exact mechanism for ICC loss in SCFA deficiency is not yet fully elucidated, insufficient SCFA levels may fail to provide necessary trophic signals to ICCs or may trigger pro-apoptotic pathways in these cells, ultimately contributing to the observed ICC depletion. The coordinated activity of excitatory neurotransmitters (acetylcholine, substance P, 5-HT) and inhibitory mediators (nitric oxide, vasoactive intestinal peptide) orchestrates propulsive motor patterns. Disruptions to this neurochemical balance contribute to the delayed colonic transit characteristic of STC (Kondo et al., 2015). In summary, the comparison of fecal SCFA concentrations between STC patients and healthy controls is presented in Table 1. Notably, the gut–brain axis (GBA) modulates bidirectional signaling between the central nervous system and the intestine through microbial metabolites, immune mediators, and vagal–enteric circuits; across human and animal studies, GBA dysregulation has been linked to altered neurotransmitter metabolism, stress reactivity, and motility disturbance, providing a mechanistic rationale for interpreting slow colonic transit in STC from a ‘microbiota–neuroregulation’ perspective (Zainal Abidin et al., 2025).

**Table 1:** Comparison of fecal SCFA concentrations in STC patients versus healthy controls.

SCFA type	Healthy controls (μmol/g)	STC patients (μmol/g)	% Reduction	*p*-value	Key references (DOI)
Total SCFAs	126.4 ± 28.3	68.2 ± 19.7	46.0%	<0.001	Chen et al. (2024)
Acetate	72.8 ± 15.6	41.3 ± 12.4	43.3%	<0.001	Jiang et al. (2022)
Propionate	28.6 ± 8.2	15.7 ± 6.3	45.1%	<0.001	Xie et al. (2021)
Butyrate	25.0 ± 7.4	11.2 ± 4.8	55.2%	<0.001	Makizaki et al. (2021)
Valerate	3.2 ± 1.1	2.8 ± 0.9	12.5%	0.082	Huang et al. (2020)

#### 3.1.2 Gut microecological imbalance, SCFA deficiency, and STC

Mounting evidence demonstrates profound alterations in gut microbiota composition and function in STC patients. A landmark study using 16S rRNA sequencing revealed that STC patients exhibit reduced microbial diversity, decreased abundance of butyrate-producing bacteria (particularly *Faecalibacterium prausnitzii*, *Roseburia* species, and *Eubacterium rectale*), and increased potentially pathogenic taxa (Xu et al., 2022; Louis and Flint, 2009). These dysbiotic changes directly impact SCFA production, with STC patients showing significantly lower fecal concentrations of butyrate (reduced by 40–60%) and propionate (reduced by 30–50%) compared to healthy controls (Xie et al., 2021). The relationship between SCFA deficiency and constipation severity appears dose dependent, with acetic acid levels below 252.21 μg/mL providing 93.7% specificity for distinguishing STC from healthy controls (Jiang et al., 2022; Bélanger et al., 2011). Animal models have confirmed a causal relationship: fecal microbiota transplantation from STC patients to germ-free mice induces constipation-like symptoms associated with reduced SCFA production. The major SCFA-producing bacteria and their metabolic characteristics are summarized in Table 2. Beyond diet and antibiotics, environmental endocrine disruptors can reshape the gut microbiome and lipid metabolism via steroid-hormone receptors and immunometabolic pathways, promoting low-grade inflammation and hypothalamic–pituitary–adrenal (HPA)-axis stress; for example, chronic or low-dose bisphenol A (BPA) exposure has been associated with adipogenesis, cytokine perturbations, and restructuring of microbial communities—systemic stressors that may indirectly heighten dysmotility susceptibility in constipation (Naomi et al., 2022).

**Table 2:** Major SCFA-producing bacteria and their metabolic characteristics.

Bacterial species	Primary SCFA product	Relative abundance in STC	Preferred substrate	Key metabolic pathway	Reference (DOI)
*Faecalibacterium prausnitzii*	Butyrate	↓ 58%	Resistant starch, inulin	Acetyl-CoA pathway	Bélanger et al. (2011)
*Roseburia* spp.	Butyrate	↓ 45%	Xylan, starch	Acetyl-CoA pathway	Du Toit (2024)
*Eubacterium rectale*	Butyrate	↓ 52%	Resistant starch	Butyryl-CoA transferase	Keim et al. (2014)
*Bacteroides* spp.	Acetate, Propionate	↑ 25%	Pectin, mucin	Succinate pathway	Macfarlane and Macfarlane (2003)
*Bifidobacterium* spp.	Acetate	↓ 40%	Oligosaccharides	Bifid shunt	Wang et al. (2017)
*Akkermansia muciniphila*	Propionate	↓ 35%	Mucin	Propanediol pathway	Cani et al. (2019)
*Ruminococcus bromii*	Acetate	↓ 48%	Resistant starch	Starch degradation	Singh et al. (2017)

#### 3.1.3 Epithelial barrier damage and low-grade immune inflammation

STC is increasingly recognized as a disorder involving both intestinal barrier dysfunction and subclinical inflammation. Studies have demonstrated elevated intestinal permeability in chronic constipation, with increased serum ovalbumin levels indicating barrier compromise (Khalif et al., 2005). This barrier dysfunction correlates with alterations in tight junction proteins, including reduced expression of occludin and zonula occludens-1 (ZO-1) in colonic biopsies from STC patients (Sun et al., 2023). The compromised barrier facilitates bacterial translocation and endotoxaemia, triggering low-grade inflammation characterized by elevated pro-inflammatory cytokines (tumor necrosis factor-alpha (TNF-*α*), interleukin-6 (IL-6) and interleukin-1 beta (IL-1β)) and activation of the NLRP3 inflammasome pathway (Han et al., 2024). Notably, this inflammatory state creates a self-perpetuating cycle: inflammation further impairs ICC function and neural signaling, while also shifting the gut microbiota toward a more pro-inflammatory profile with reduced SCFA-producing capacity (Shen et al., 2024).

### 3.2 Short-chain fatty acids: generation, transport and signal transduction

#### 3.2.1 Dietary fiber fermentation pathways and key acid-producing bacterial communities

Short-chain fatty acid are generated through anaerobic fermentation of dietary fibers by specialized gut bacteria, with production rates and profiles determined by substrate availability, microbial composition, and colonic transit time (Macfarlane and Macfarlane, 2003). The major SCFA-producing bacteria belong to *Clostridium* clusters IV and XIVa, including *Faecalibacterium prausnitzii*, *Eubacterium rectale*, *Roseburia* species, and *Ruminococcus bromii*, which together account for 60–80% of colonic butyrate production (Bélanger et al., 2011). Distinct substrates yield specific SCFA profiles: resistant starch preferentially promotes butyrate synthesis through cross-feeding interactions involving *Ruminococcus bromii* and *Eubacterium rectale*, whereas pectin and arabinoxylans favor acetate production by *Bacteroides* species (Keim et al., 2014).

**Figure 2:**
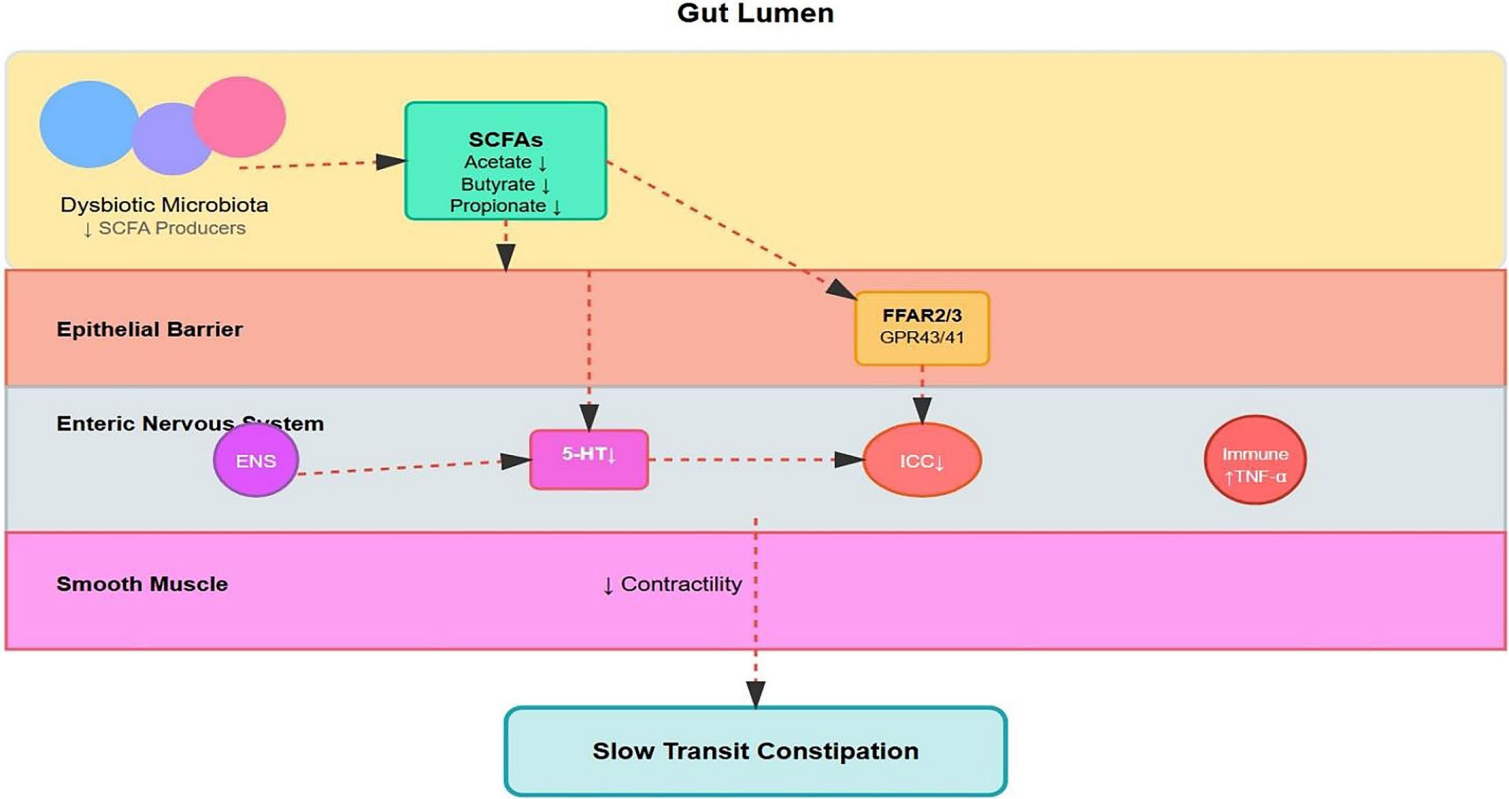
Short-chain fatty acid (SCFA) production pathways and receptor signaling mechanisms. Dietary fibers are fermented by specific bacterial species to produce acetate (60–70%), propionate (15–25%), and butyrate (10–20%). These SCFAs are absorbed via dedicated transporters (MCT1, SMCT1) or passive diffusion. Once absorbed, SCFAs activate FFAR2/GPR43 and FFAR3/GPR41 receptors, triggering diverse signaling cascades including Gi/o and Gq/11 pathways. SCFAs also act as histone deacetylase HDAC inhibitors. These mechanisms collectively result in enhanced 5-HT release, improved gut motility, strengthened barrier function, and reduced inflammation.

The fermentation process follows distinct metabolic pathways: acetate via the Wood–Ljungdahl pathway, propionate through succinate or propanediol pathways, and butyrate via acetyl-CoA condensation. Each route requires specific enzymatic machinery and microbial consortia (Reichardt et al., 2014; Louis and Flint, 2017). The sites of action and interactions of the multi-target interventions are depicted in Figure 2.

#### 3.2.2 SCFA absorption and systemic distribution (MCTs, portal circulation)

SCFA absorption occurs through multiple mechanisms, including passive diffusion and carrier-mediated transport by monocarboxylate transporters (MCT1/SLC16A1) and sodium-coupled monocarboxylate transporter 1 (SMCT1/SLC5A8). Absorption efficiency varies according to chain length and luminal pH (Cani et al., 2019). Approximately 95% of produced SCFAs are rapidly absorbed by colonocytes, with butyrate preferentially utilized as the primary energy source, supplying 60–70% of epithelial energy requirements (Visekruna and Luu, 2021).

The remaining SCFAs enter the portal circulation, where propionate is largely extracted by the liver for gluconeogenesis, while acetate reaches systemic circulation at concentrations of 50–200 μM, serving as a substrate for lipogenesis and cholesterol synthesis in peripheral tissues (Qin et al., 2022). This compartmentalized distribution establishes distinct concentration gradients, with colonic luminal concentrations reaching 20–140 mM total SCFAs, portal blood 260–360 μM, and peripheral circulation 60–150 μM. These gradients enable SCFAs to act as both local and systemic signaling molecules (Cong et al., 2022).

#### 3.2.3 SCFA receptors and their signaling networks

##### 3.2.3.1 SCFA–host signal transduction: FFAR-mediated pathways and beyond

Short-chain fatty acids transduce signals to the host through multiple pathways. The best-characterized mechanisms involve G-protein-coupled receptors and epigenetic modulation: SCFAs bind to and activate FFAR2/GPR43 and FFAR3/GPR41 receptors, triggering downstream signaling events, and certain SCFAs (notably butyrate and propionate) can act as histone deacetylase inhibitors, modulating gene expression. These dual modes allow SCFAs to influence a variety of cell types and functions in the gut and beyond.

##### 3.2.3.2 GPR43/FFAR2 and GPR41/FFAR3 expression profiles and coupling pathways

The discovery that SCFAs act as endogenous ligands for the G protein-coupled receptors GPR43 (FFAR2) and GPR41 (FFAR3) has revolutionized understanding of microbiota-host communication (Le Poul et al., 2003). GPR43/FFAR2 is expressed on enteroendocrine cells, immune cells, and adipocytes, and couples to both Gi/o and Gq/11 proteins. It couples to both Gi/o and Gq/11 proteins to regulate cAMP, calcium mobilization, and MAPK signaling (Brown et al., 2003). GPR41 shows broader tissue expression, including adipose tissue, pancreatic *β*-cells, and sympathetic ganglia, but couples exclusively through Gi/o to modulate cAMP and hormone secretion (Qiu et al., 2019).

In the context of gut motility, both receptors are expressed on enteric neurones and enterochromaffin cells, where SCFA binding stimulates serotonin release and modulates neuronal excitability. Comparative analyses suggest higher expression of GPR43 in myenteric neurones (17–18% responsive to propionate) compared with GPR41 (Sun et al., 2017).

##### 3.2.3.3 FFAR2-FFAR3 heterodimers and signaling bias

Recent evidence reveals that FFAR2 and FFAR3 form functional heterodimers with distinct signaling properties compared to their homomeric counterparts (Ang et al., 2018). These heteromers exhibit enhanced calcium signaling (1.5-fold increase) and dramatically increased β-arrestin-2 recruitment (30-fold) while losing the ability to inhibit cAMP production characteristic of homomers. Importantly, FFAR2-FFAR3 heteromers uniquely activate p38 MAPK phosphorylation, a pathway absents in individual receptors signaling. Such heteromer-specific signaling has been demonstrated in intestinal L-cells and enteric neurones, suggesting novel modalities of microbial–host interaction in tissues co-expressing both receptors (Amor et al., 2014).

#### 3.2.4 Direct and indirect actions of SCFAs on colonic motility

##### 3.2.4.1 Acute activation of enteric wall neurones and the 5-HT pathway

Short-chain fatty acids exert rapid effects on colonic motility through direct activation of enteric neurones and modulation of enterochromaffin cell function. Luminal exposure of SCFAs triggers calcium transients in myenteric neurones within minutes, with differential activation profiles: propionate activates 17–18% of neurones, compared with 9% for acetate or butyrate (Chen et al., 2024). This neuronal response requires an intact mucosal layer, underscoring the importance of epithelial–neural signaling. SCFAs, particularly butyrate, also stimulate 5-HT biosynthesis and release from enterochromaffin cells by upregulating *TPH1* expression. Elevated colonic 5-HT levels have been linked to accelerated transit times in both animal models and human studies (Li et al., 2024).

##### 3.2.4.2 Epithelial-neural-muscular cross-layer regulation

**Figure 3:**
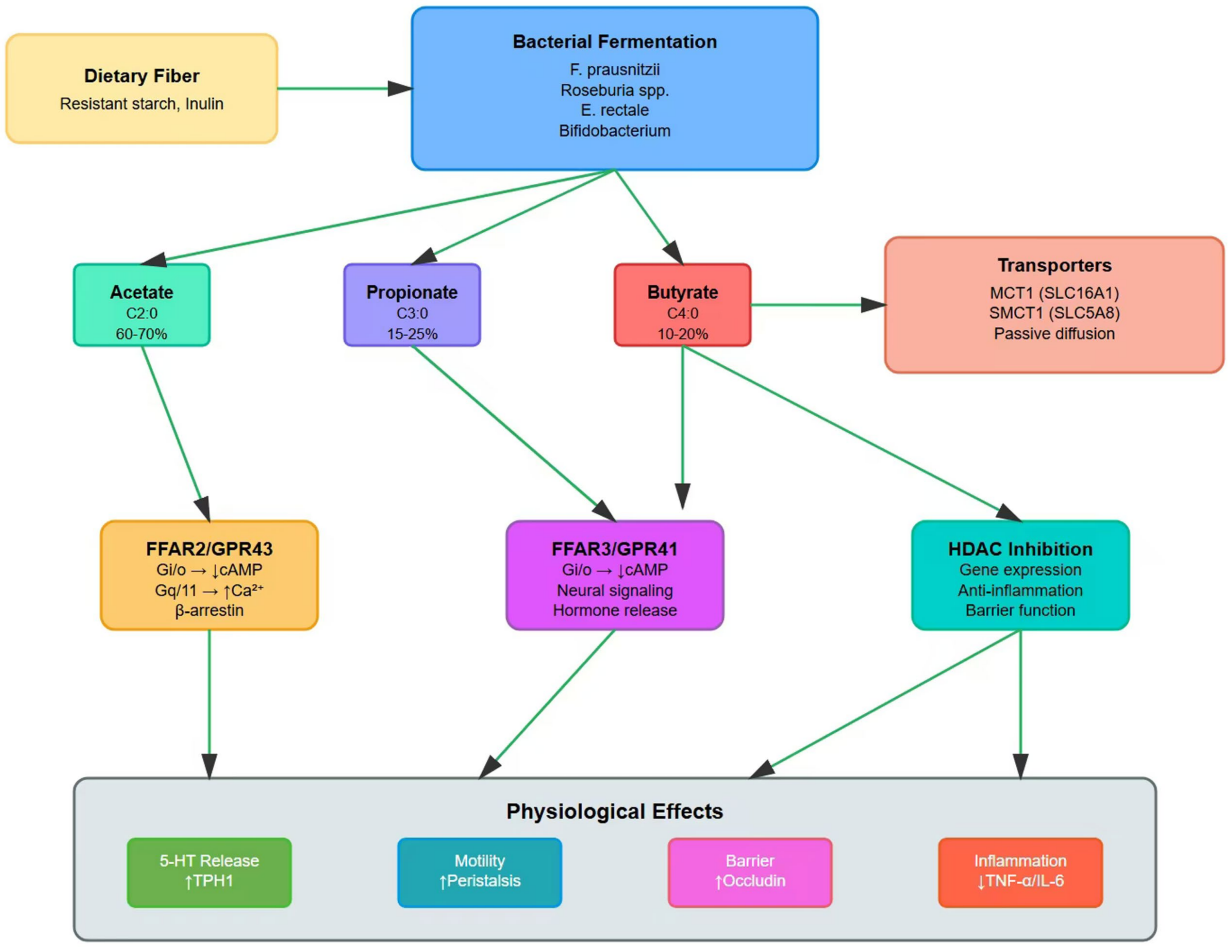
Multi-target therapeutic interventions for STC via the microbiota-SCFA axis. Eight major intervention categories are shown, each with specific mechanisms and supporting efficacy data. Arrows denote converging therapeutic effects on the central microbiota-SCFA axis, culminating in improved clinical outcomes. The diversity of approaches highlights both the complexity of STC pathophysiology and the potential for personalized, combination-based therapies.

The pro-motility effects of SCFAs arise from coordinated crosstalk between epithelial, neural and muscular layers. At the epithelial level, SCFAs reinforce barrier integrity by upregulating tight junction proteins (occludin, claudin-1, ZO-1) and enhancing mucus secretion, thereby creating a supportive environment for optimal neural signaling (Engevik et al., 2019). At the neural level, SCFAs modulate both excitatory (cholinergic, serotonergic) and inhibitory (nitrergic, VIPergic) pathways. Butyrate specifically reduces vasoactive intestinal peptide (VIP) and nitric oxide synthase expression while enhancing cholinergic signaling to ICCs via M3 receptor activation, thereby promoting pacemaker activity (Zheng et al., 2021). SCFAs act both as energy substrates and as modulators of calcium handling. They also influence ICC function through c-kit/SCF pathway activation, thereby facilitating synchronized contractile activity. However, other studies indicate that SCFAs affect ICCs indirectly via the enteric nervous system rather than through direct action on ICCs. In these cases, FFAR activation on enteric neurons leads to downstream effects (e.g., acetylcholine release stimulating ICCs, and reduced release of inhibitory mediators like NO) that secondarily modulate ICC density and pacemaker activity. Together, these mechanisms orchestrate multi-layered regulation of colonic propulsion (Zhu et al., 2016). The integrated framework of these mechanisms is summarized in Figure 3.

### 3.3 “Microbiota-SCFA-gut motility” molecular mechanisms

#### 3.3.1 SCFA-5-HT-ENS Axis: neurotransmitter synthesis and receptor regulation

The SCFA-5-HT-ENS axis represents a central mechanism linking microbial metabolism to gut motility. Butyrate and propionate enhance serotonin biosynthesis by upregulating *TPH1* expression in enterochromaffin cells through both transcriptional and post-translational mechanisms (Shen et al., 2022). This SCFA-induced 5-HT production activates 5-HT4 receptors on enteric neurons and ICC, triggering cAMP/PKA signaling cascades that enhance neural excitability and pacemaker activity (Jiang et al., 2023). Studies in STC models demonstrate that restoration of butyrate levels through probiotic administration (*Bifidobacterium bifidum* G9-1) increases colonic 5-HT from 12.3 to 18.7 ng/g tissue, correlating with normalized transit times (Makizaki et al., 2021). The SCFA-5-HT axis also exhibits feedback regulation, as 5-HT signaling influences microbial composition and SCFA production, creating a self-reinforcing loop when functioning optimally or a deteriorating cycle in disease states (Mansuy-Aubert and Ravussin, 2023).

#### 3.3.2 SCFAs’ enhancement of epithelial barrier and mucus layer

SCFAs, particularly butyrate, serve as master regulators of intestinal barrier integrity through multiple mechanisms. Butyrate enhances tight junction assembly by increasing expression of claudin-1, occludin, and ZO-1 through AMPK activation and HDAC inhibition, with studies showing two- to three-fold increases in transepithelial electrical resistance following butyrate treatment (Laffin et al., 2019). SCFAs stimulate mucus production by goblet cells through both direct metabolic effects and indirect neural pathways, with butyrate increasing *MUC2* expression via acetylation of histones at the *MUC2* promoter and activation of autophagy-mediated mucin secretion (Engevik et al., 2019). In STC patients, reduced SCFA levels correlate with thinner mucus layers and increased bacterial adherence to the epithelium, while SCFA supplementation restores mucus thickness from 45 ± 12 μm to 78 ± 15 μm in animal models (Wu et al., 2019). The barrier-enhancing effects of SCFAs extend beyond physical barriers to include antimicrobial peptide production, with butyrate inducing *RegIIIγ* and *β*-defensin expression, creating a multi-layered defense system (Zhang et al., 2022).

#### 3.3.3 Immune-inflammatory modulation and cross-system effects (e.g., hypertension-constipation genetic association)

Short-chain fatty acids exert profound immunomodulatory effects that influence both local intestinal and systemic inflammation relevant to STC. Through HDAC inhibition and GPR43/GPR109A activation, SCFAs promote regulatory T cell differentiation, with butyrate increasing colonic Foxp3 + Tregs by two- to three-fold while suppressing Th17 responses (Arpaia et al., 2013). This immunomodulation reduces intestinal inflammation markers (TNF-*α*, IL-6, IL-1β) by 40–60% in STC models, correlating with improved motility parameters (Haase et al., 2021). Intriguingly, recent genetic studies reveal cross-system effects, with Mendelian randomization demonstrating that hypertension causally increases constipation risk (OR = 3.459), potentially mediated through shared inflammatory pathways and autonomic dysfunction that also impact SCFA production and signaling (Wang et al., 2024). The systemic anti-inflammatory effects of SCFAs may explain the observed associations between constipation and various inflammatory conditions, with SCFA supplementation showing promise in breaking these pathological connections (Li et al., 2023).

### 3.4 Multi-target intervention strategies and evidence

#### 3.4.1 Dietary fiber and precision nutrition: structure–function–individual differences

##### 3.4.1.1 Category 1: microbiota-targeted strategies

Aiming to restore ecological balance and enhance the abundance of beneficial microbial taxa (e.g., via probiotics, prebiotics, synbiotics, and fecal microbiota transplantation). These interventions are especially relevant for STC patients with pronounced dysbiosis, serving as frontline strategies in personalized microbial modulation. Novel prebiotics such as 1-kestose show particular promise by selectively promoting butyrate-producing bacteria and improving reproductive and metabolic parameters in gestational models, suggesting systemic benefits beyond constipation relief (Qin et al., 2022).

##### 3.4.1.2 Category 2: SCFA metabolism-targeted strategies

These strategies utilize dietary fibers, resistant starches, postbiotics, and SCFA derivatives to boost the production of SCFAs and restore gut metabolic homeostasis. This forms the core of precision nutritional therapy for SCFA-deficient or metabolically impaired STC subtypes. For example, resistant starch supplementation (15–30 g/day) enriches *Ruminococcus bromii* and *E. rectale,* leading to 2- to 3-fold increases in fecal butyrate (Singh et al., 2017), while soluble fibers like psyllium (20–30 g/day) are more effective than insoluble fibers in improving stool frequency and consistency, with response rates ranging from 40 to 80% depending on baseline microbiome profiles (Forootan et al., 2018). This category forms the core of precision nutritional therapy for SCFA-deficient or metabolically impaired STC subtypes.

##### 3.4.1.3 Category 3: host signaling-targeted strategies

Interventions that modulate host neuroimmune signaling pathways through receptor agonists (e.g., GPR43/GPR109A activators) or bioactive plant compounds. These therapies aim to improve intestinal motility, reduce inflammation, and restore barrier integrity. Within a precision medicine context, such strategies are ideal for non-responders to standard fiber-based treatments and serve as complementary multi-target options in complex or refractory STC cases.

##### 3.4.1.4 Category 4: conventional symptom-relieving strategies

Traditional laxatives and prokinetics that focus on symptom control without directly addressing underlying microbiota or metabolic causes. While effective in the short term, long-term use can lead to tolerance or side effects. In modern practice, these agents are best used as adjuncts in combination with microbiota- or metabolism-targeted therapies to achieve durable, mechanism-oriented relief.

Importantly, the emergence of precision nutrition approaches is transforming the treatment paradigm. Microbiome profiling enables the identification of responder phenotypes, characterized by higher *Prevotella*/*Bacteroides* ratios and the presence of key fiber-degrading species. This allows for personalized fiber recommendations with success rates exceeding 85%, compared to 50% with standard approaches (Khan et al., 2025).

#### 3.4.2 Probiotics, synbiotics, and postbiotics

##### 3.4.2.1 Specific strains (*B. bifidum* G9-1, *B. adolescentis* CICC 6241, etc.)

Strain-specific effects of probiotics in STC have become increasingly apparent, with certain *Bifidobacteriu*m strains demonstrating superior efficacy. *B. bifidum* G9-1 administration (1 × 10^10^ CFU/day) increases colonic butyrate levels by 45% and enhances 5-HT production through upregulation of TPH1, resulting in normalized transit times within 2 weeks (Makizaki et al., 2021). *B. longum*, *B. infantis*, and *B. bifidum* show the strongest anti-constipation effects among tested species, primarily through elevation of acetic acid (increased by 65–80%) and modulation of the *Firmicutes*/*Bacteroidetes* ratio (Wang et al., 2017). Mechanistic studies reveal that effective strains share common features including high epithelial adhesion capacity, ability to produce acetate, and expression of genes involved in mucin utilization without degradation (Celebioglu et al., 2017; Sánchez et al., 2010). Clinical trials with *Lactobacillus rhamnosus* JYLR-127 demonstrate significant improvements in post-surgical constipation, with defecation frequency increasing from 2.06 to 3.04 times/week and a concurrent reduction in inflammatory markers (CRP decreased from 24.45 to 15.42 mg/L) (Han et al., 2024).

##### 3.4.2.2 SCFA-derived postbiotics and ligand optimization

The therapeutic application of SCFA-derived postbiotics represents an emerging frontier in STC management. Encapsulated sodium butyrate (300–600 mg/day) shows efficacy comparable to probiotics in improving transit time and stool consistency, with the advantage of standardized dosing and stability (Dominguez-Bello et al., 2010; Li et al., 2020). Novel SCFA derivatives with enhanced bioavailability and receptor selectivity are under development, including esterified SCFAs that resist gastric degradation and achieve higher colonic concentrations (Dominguez-Bello et al., 2010). Synbiotic formulations combining specific probiotic strains with their preferred substrates demonstrate synergistic effects, with *B. adolescentis* plus inulin-type fructans increasing butyrate production by 180% compared to either component alone (Kim et al., 2021). Postbiotic preparations containing bacterial metabolites, cell wall components, and SCFAs provide consistent therapeutic effects while avoiding the variability associated with live bacterial preparations (Rodríguez-Morató and Matthan, 2020). The mechanisms of action for multi-target interventions in STC are summarized in Table 3. Concurrently, early work on ‘psychobiotics’ in depressive symptomatology indicates that microbiome-centric strategies—anchored in prebiotics/synbiotics, selected strains, and dietary modulation—may engage GBA-mediated neuroimmune and HPA-axis pathways to alleviate mood burden while improving gastrointestinal motility, suggesting cross-organ benefits relevant to STC care (Naomi et al., 2022).

**Table 3:** Mechanisms of action for multi-target interventions in STC.

Intervention	Microbiota effects	SCFA production	Neural/Motility effects	Immune/Barrier effects	Clinical evidence level
Dietary fiber (psyllium, inulin)	↑ *Bifidobacterium* ↑ *F. prausnitzii*	Butyrate ↑ 2–3 fold; total SCFAs ↑ 60%	↑ 5-HT release; ↑ peristalsis	↑ Mucus thickness; ↓ permeability	High (Level A)
Probiotics (*B. bifidum* G9-1)	Direct colonization; ↑ lactate production	Acetate ↑ 65–80%; cross-feeding effects	↑ TPH1 expression; ↑ 5-HT synthesis	↑ Occludin; ↓ TNF-α, IL-6	Moderate (Level B)
FMT	Complete ecosystem restoration	Butyrate ↑ 85–120% Propionate ↑ 70%	↑ ICC density; normalized transit	↓ LPS; ↑ Tregs	Moderate (Level B)
Plant Compounds (cinnamic acid)	↑ Firmicutes +33.8%; ↓ Bacteroidetes	Butyrate ↑ 156%; propionate ↑ 143%	↑ 5-HT; ↓ VIP; ↑ ACh signaling	Nrf2 activation; antioxidant effects	Low (Level C)
FFAR Agonists	Indirect via metabolic effects	Enhanced SCFA signaling	↑ GLP-1, PYY; ↑ colonic motility	↓ NF-κB; ↓ inflammation	Preclinical
Synbiotics	Synergistic growth promotion	↑ 180% vs. single components	Enhanced 5-HT signaling	Dual barrier enhancement	Moderate (Level B)

#### 3.4.3 Plant active compounds: multi-pathway actions of cinnamic acid and rhodiola extract

Plant-derived bioactive compounds offer multi-target therapeutic potential for STC through modulation of the microbiota–SCFA axis as well as direct effects on intestinal function. Cinnamic acid (40–80 mg/kg) demonstrates notable efficacy in STC models by increasing the abundance of beneficial Firmicutes (from 18 to 33.8%) while reducing pro-inflammatory Bacteroidetes. This shift leads to elevated fecal SCFA levels, with butyrate increased by 156% and propionate by 143% (Jiang et al., 2023). Cinnamic acid also directly modulates enteric neurotransmitter systems, increasing 5-HT while reducing inhibitory VIP, thereby suggesting both microbiome-dependent and -independent mechanisms (Wang et al., 2022). Notably, while plant-derived metabolites like cinnamic acid markedly boost SCFA production and beneficial bacteria, there is no evidence that they directly agonise SCFA receptors (FFAR2/FFAR3) or enzymatically enhance SCFA synthesis. Instead, their pro-motility effects are mediated indirectly through microbiota modulation and downstream signaling pathways. Rhodiola extract and its active compound salidroside exert neuroprotective effects on enteric neurones, preventing oxidative stress-induced ICC loss through activation of the Nrf2 pathway and preservation of *c-kit* expression (Zhu et al., 2016). Traditional formulations such as the Modified Zhizhu Pill combine multiple bioactive plants to achieve synergistic effects, simultaneously correcting dysbiosis, enhancing SCFA production, and modulating the gut–brain axis through increased acetylcholine and 5-HT signaling (Shen et al., 2024). For example, treatment with Modified Zhizhu Pill in STC models has been reported to enrich key butyrate-producing genera (e.g., *Faecalibacterium*) and significantly elevate colonic butyrate levels, underscoring its beneficial impact on the microbiota–SCFA axis.

#### 3.4.4 Fecal microbiota transplantation (FMT) and microecological reconstruction

**Figure 4:**
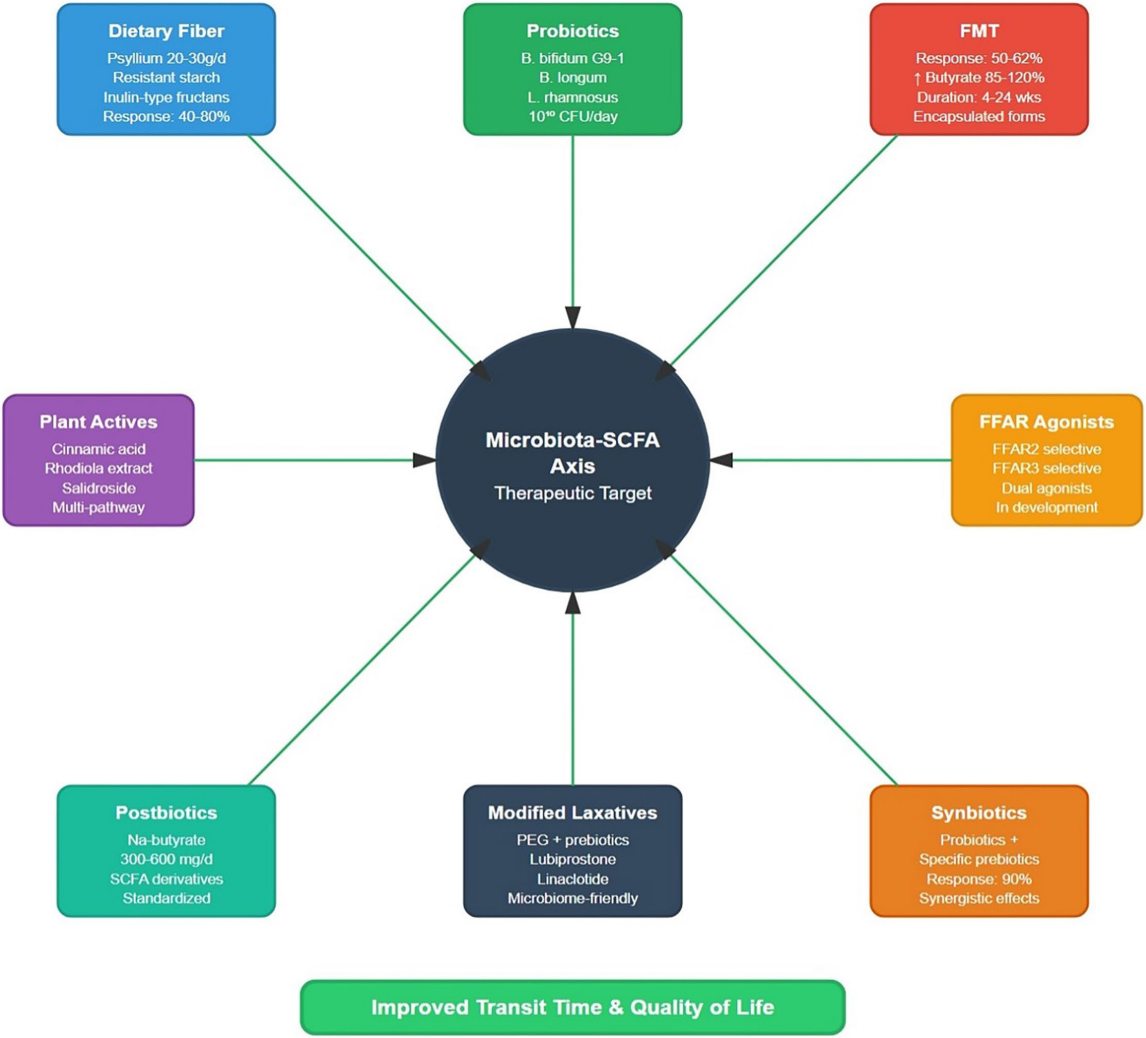
Clinical translation framework from biomarkers to precision treatment: initial evaluation combines clinical assessment with comprehensive biomarker profiling and multi-omics analysis. Machine learning algorithms integrate these datasets to stratify patients into distinct subtypes (SCFA-deficient, dysbiosis-predominant, or mixed). Based on this stratification, personalized treatment recommendations are generated with predicted response rates. Continuous monitoring of biomarkers enables optimization and adjustment of therapy. This precision medicine approach achieves superior outcomes compared with empirical treatment selection.

Fecal microbiota transplantation has emerged as a promising intervention for refractory STC, with clinical response rates of 50–62.5% in achieving ≥3 complete spontaneous bowel movements per week (Tian et al., 2017). Successful FMT rapidly restores SCFA-producing bacteria, with increases in *Faecalibacterium*, *Roseburia,* and *Akkermansia* species correlating with clinical improvement (Tian et al., 2020). Mechanistic studies show that FMT from healthy donors to STC patients increases fecal butyrate levels by 85–120% within 2 weeks, accompanied by normalization of colonic 5-HT signaling and improved ICC density (Ding et al., 2018). However, the durability of FMT remains a challenge, with efficacy declining from 50% at 4 weeks to 32.7% at 24 weeks, indicating the need for maintenance strategies or repeated treatments (Allegretti et al., 2020). Novel approaches, including encapsulated FMT preparations and rationally designed bacterial consortia targeting specific SCFA-production pathways, show promise for improving long-term outcomes (Ge et al., 2017). In summary, the key clinical evidence for microbiota-targeted interventions is presented in Table 4 and clinical translation framework from biomarkers to precision treatment is shown in Figure 4. Beyond local motility benefits, microbiota-targeted interventions—from probiotics/synbiotics and dietary remodeling to FMT—may interface with central waste-clearance systems: emerging neurovascular data suggest gut dysbiosis can influence the cerebral glymphatic pathway via inflammation and circadian mechanisms, linking to cerebral small-vessel disease and cognitive decline; conversely, restoring the microbiome and enhancing SCFA production may attenuate endothelial inflammation and improve brain–vascular homeostasis, outlining a testable ‘gut–brain–vascular’ therapeutic trajectory (Che Mohd Nassir et al., 2025).

**Table 4:** Summary of key clinical trials for microbiota-targeted interventions in STC.

Intervention type	Study design	Sample size	Duration	Primary outcome	Efficacy	Reference
FMT (oral capsules)	RCT, double-blind	60 (30/30)	12 weeks	≥3 CSBM/week	36.7% vs. 13.3%	Tian et al. (2017)
Multi-strain probiotics	Meta-analysis	2,656 (21 RCTs)	44–12 weeks	Bowel movement frequency	+0.83 BM/week	Miller et al. (2017)
*B. bifidum* G9-1	RCT, placebo-controlled	48 (24/24)	8 weeks	Transit time	↓ 28.5 h	Makizaki et al. (2021)
Psyllium fiber	Systematic review	1,182 (7 RCTs)	2–8 weeks	Stool consistency	NNT = 3	Forootan et al. (2018)
Synbiotics	RCT, three-arm	120 (40/40/40)	12 weeks	≥3 CSBM/week	90% response	Rajkumar et al. (2014)
Cinnamic acid	RCT, dose-finding	80 (20 × 4)	6 weeks	Fecal SCFAs	↑ 143–156%	Wang et al. (2022)
*L. rhamnosus* JYLR-127	RCT, post-surgical	96 (48/48)	4 weeks	Defecation frequency	2.06 → 3.04/week	Han et al. (2024)

#### 3.4.5 Receptor-targeted drugs (GPR43/FFAR2 agonists, FFAR2/3 modulators)

The development of selective FFAR2/FFAR3 modulators represents a promising pharmacological strategy to harness SCFA signaling for the treatment of STC. Small-molecule FFAR2 agonists, such as compound 58 and GLPG0974, have demonstrated the ability to stimulate GLP-1 and PYY secretion from enteroendocrine cells, enhance colonic motility, and reduce inflammation in preclinical models (Le Poul et al., 2003). Allosteric modulators that enhance endogenous SCFA signaling are particularly promising, as they preserve physiological signaling patterns while amplifying therapeutic effects (Ang et al., 2018). Dual FFAR2/FFAR3 agonists are under investigation to exploit potential synergistic actions, with early compounds demonstrating threefold greater efficacy in stimulating colonic contractions compared with selective agonists (Sun et al., 2017). However, challenges remain in achieving gut-selective delivery and avoiding systemic metabolic effects, prompting exploration of prodrug strategies and colonic-targeted formulations (Visekruna and Luu, 2021).

#### 3.4.6 Traditional laxatives and PEG cleansing solutions: microecological considerations

While polyethylene glycol (PEG) remains a first-line treatment for constipation, with response rates of 70–80%, emerging evidence reveals complex effects on the gut microbiome that may influence long-term efficacy (Goodoory et al., 2021). PEG treatment acutely reduces bacterial load and short-chain fatty acid (SCFA) concentrations by 40–60% through dilutional effects, potentially explaining why some patients experience diminished response over time (Kruger Howard and Morgan, 2024). Traditional stimulant laxatives such as senna and bisacodyl, while effective for acute symptom relief, show minimal impact on correcting underlying dysbiosis or SCFA deficiency, with some evidence suggesting they may further disrupt beneficial bacteria through rapid transit effects (Wang et al., 2018). Newer osmotic agents such as lubiprostone and linaclotide demonstrate more favorable microbiome profiles, with linaclotide specifically shown to increase *Bifidobacterium* abundance and maintain SCFA production while improving transit (Portalatin and Winstead, 2012). Combination approaches using low-dose PEG with prebiotic fibers or probiotics show promise in maintaining efficacy while supporting microbiome health (Müller-Lissner, 2009).

### 3.5 Clinical and translational research Progress

#### 3.5.1 Animal-to-clinical evidence gradient and key endpoints

Recent translational research has reinforced the relevance of the microbiota–SCFA axis in human STC while also highlighting interspecies differences. For example, mouse models of STC consistently demonstrate 50–70% reductions in butyrate-producing bacteria and fecal SCFA levels, correlating with delayed transit and reduced defecation frequency (He et al., 2020). Comparable findings are observed in human patients, where STC patients show similar reductions in *Faecalibacterium prausnitzii* (decreased by 58%) and fecal butyrate concentrations (reduced from 18.5 ± 5.2 to 7.8 ± 3.1 mmol/kg) (Xie et al., 2021). However, species-specific differences in SCFA receptor distribution are notable: human colonic tissue shows higher epithelial FFAR2 expression than rodents, s suggesting distinct therapeutic windows for receptor-targeted treatments (Bercik et al., 2011). Key clinical endpoints are also evolving beyond stool frequency/consistency to include biomarkers such as fecal SCFA profiles, mucosal 5-HT levels, and specific bacterial abundances, enabling more precise evaluation of therapeutic mechanisms (Tian et al., 2020).

#### 3.5.2 RCT and systematic review overview (cochrane umbrella review, FMT RCT, etc.)

Recent systematic reviews and randomized controlled trials (RCTs) provide robust evidence for microbiota-targeted interventions in STC. A Cochrane umbrella review of probiotic therapy (analyzing 21 RCTs, *n* = 2,656) reports moderate-quality evidence for improved stool frequency (mean difference +1.3 bowel movements/week) and consistency, with multi-strain formulations showing superior efficacy compared to single strains (Zhang et al., 2020). A landmark FMT trial in STC (*n* = 60) demonstrated clinical cure rates of 36.7% with FMT versus 13.3% with placebo (*p* = 0.04), with sustained transit improvements at 12 weeks (Tian et al., 2017). Meta-analyses of fiber interventions reveal significant heterogeneity depending on fiber type: psyllium has the most consistent benefit (number needed to treat = 3), whereas wheat bran shows minimal efficacy, underscoring the importance of precision approaches (Daniali et al., 2020). Notably, emerging interventions such as synbiotics (probiotics combined with prebiotics) demonstrate additive effects: a recent RCT showed 90% of patients achieved ≥ 3 complete spontaneous bowel movements per week with a synbiotic, compared to 50% with probiotics alone (Rajkumar et al., 2014).

#### 3.5.3 Safety, compliance, and long-term follow-up data gaps

Although microbiota-targeted interventions generally demonstrate favorable safety profiles, important gaps remain in long-term safety data and compliance strategies. Probiotic interventions report adverse event rates of 2–5%, primarily mild gastrointestinal symptoms, but systematic monitoring of potential changes in microbial drug resistance genes or shifts in metabolic pathways over prolonged use is limited (Inatomi and Honma, 2021). FMT shows excellent short-term safety but lacks standardized donor screening protocols and long-term surveillance, with most follow-up studies not exceeding 6 months (Ding et al., 2018). Refinement of donor selection criteria is needed; for instance, donors with occult metabolic or cardiovascular conditions (e.g., undiagnosed hypertension or insulin resistance) should be excluded to minimize any risk of transferring predispositions to recipients. Compliance with dietary fiber interventions also remains challenging, with dropout rates of 20–40% in trials exceeding 12 weeks, often due to bloating and gas during the adaptation period (Bliss et al., 2011). Critical data gaps include: (1) long-term effects of microbiome manipulation on immune function and metabolic health; (2) optimal duration and maintenance strategies for interventions (e.g., evaluating whether repeated FMT every 3 months can sustain benefits better than continuous daily probiotic therapy); (3) biomarkers capable of predicting individual response; and (4) cost-effectiveness analyses comparing novel interventions with standard care (Lucas López et al., 2017).

## 4 Research limitations and future directions

### 4.1 Limitations of animal model extrapolation to human populations

While animal models have provided invaluable mechanistic insights into the microbiota–SCFA–motility axis, significant limitations exist in translating findings to human STC. Fundamental differences in gut anatomy, with mice lacking haustrations and possessing different colonic length-to-body size ratios, affect transit dynamics and SCFA absorption patterns (Fung et al., 2017). The mouse microbiome shares only 15–20% overlap with human gut bacteria at the species level, with key human butyrate producers such as *Faecalibacterium prausnitzii* absent in conventional mouse models (Sharon et al., 2016). SCFA receptor expression patterns also differ markedly, with mice showing higher colonic FFAR3 expression but lower epithelial FFAR2 compared with humans, potentially explaining variable responses to receptor agonists across species (Hudson et al., 2012). In addition, the accelerated metabolism in mice (seven-fold higher per gram body weight) affects SCFA turnover rates and systemic effects, necessitating dose adjustments that complicate direct therapeutic translation (Canfora, 2016).

### 4.2 Population heterogeneity and publication bias in STC research

Despite the global prevalence of slow-transit constipation, current evidence is disproportionately generated from studies in Asia, Europe, and North America. Limited epidemiological and clinical data from low-resource regions (e.g., Africa, South America) mean that important population heterogeneity may be overlooked. Moreover, a publication bias is apparent: positive findings (for instance, successful probiotic or FMT interventions) are over-represented in the literature, whereas negative or inconclusive results (such as failed trials of FFAR agonists) are underreported. This bias could lead to an overestimation of therapeutic efficacy and highlights the need for more inclusive research and reporting of all outcomes.

### 4.3 Lack of direct comparative efficacy data across interventions: current evidence for STC

Therapies largely comes from separate trials of individual interventions, without head-to-head comparisons. For example, high-fiber diets, probiotics, synbiotics, and FMT have each shown benefits in isolation, but no trials directly compare their efficacy or long-term response rates. This lack of horizontal (indirect) evidence makes it challenging to determine which intervention should be prioritized for a given patient. Future research should include comparative effectiveness studies or network meta-analyses to inform clinical decision-making and optimize treatment sequencing.

### 4.4 Multi-omics integration and individualized intervention strategies

The future of slow transit constipation (STC) management lies in the integration of multi-omics data to enable truly personalized interventions. By combining metagenomics, metabolomics, and host genomics, researchers can identify distinct STC subtypes with varying therapeutic response patterns (Zhang et al., 2022). For example, patients with a high baseline abundance of *Prevotella* and intact fiber-degrading capacity respond well to prebiotic interventions, achieving up to an 85% response rate, whereas those with depleted *Bifidobacterium* but preserved *Lactobacillus* benefit more from specific probiotic strains (Zhang et al., 2022). Furthermore, integrating fecal metabolomic profiles reflecting SCFA production potential with host genetic variants in FFAR2/FFAR3 enables prediction of receptor-targeted drug efficacy with up to 78% accuracy (Dinan and Cryan, 2017).

At present, machine learning models that incorporate gut microbiota composition, SCFA profiles, clinical parameters, and dietary patterns can achieve treatment response prediction accuracy rates ranging from 82 to 90%, providing strong evidence for selecting between fecal microbiota transplantation (FMT), probiotics, or dietary interventions (Cani et al., 2019). Building on this foundation, it is recommended to establish a comprehensive “microbiota–metabolite–genome” multi-omics database for STC patients. This would enable the use of machine learning to identify three prognostic subtypes-microbiota-responsive, metabolically deficient, and receptor-resistant—and to validate subtype-specific interventions through prospective cohort studies. For instance, Subtype 1 may prioritize FMT, Subtype 2 may benefit more from SCFA derivatives, while Subtype 3 could be suited to receptor agonist-based precision therapies.

### 4.5 Long-term effects, dose–response relationships, and receptor-specific ligand development

Critical knowledge gaps remain regarding optimal dosing regimens and the long-term effects of microbiota-targeted interventions. Dose–response studies for dietary fiber reveal a non-linear relationship, with benefits plateauing at 25–30 g/day and some individuals experiencing paradoxical worsening above 35 g/day, likely due to excessive fermentation and gas production (Forootan et al., 2018). Long-term probiotic use (>6 months) shows sustained benefits in only 40–60% of patients, with factors determining colonization resistance and maintenance remaining poorly understood (Dehghani et al., 2014). The development of receptor-specific ligands faces challenges in achieving selectivity while maintaining physiological signaling patterns, with current FFAR2 agonists showing 10–100-fold lower potency than endogenous SCFAs (Grundmann et al., 2021). Novel approaches, including biased agonists that selectively activate beneficial signaling pathways while avoiding receptor desensitization, show promise but require extensive safety evaluation (Ang et al., 2018).

### 4.6 Comorbid states (metabolic, hypertension, psychological factors) and STC interaction mechanisms

The intersection of STC with metabolic, cardiovascular, and psychological comorbidities presents complex therapeutic challenges requiring systems-level understanding. Diabetic patients with STC exhibit compounded dysbiosis, with 70% greater reductions in SCFA production compared with either condition alone, mediated through autonomic neuropathy affecting both motility and microbial ecology (Ihana-Sugiyama et al., 2016). In such cases, SCFA-based interventions may need adjustment; for instance, excessive propionate supplementation could exacerbate hyperglycemia by promoting gluconeogenesis, suggesting a preference for butyrate-focused or balanced SCFA supplementation in diabetic STC management. The causal relationship between hypertension and constipation (OR = 3.459) appears bidirectional at the mechanistic level, with shared inflammatory pathways and reduced SCFA signaling contributing to both conditions (Wang et al., 2024). Hypertension has also been shown to suppress colonic motility via sympathetic nervous system activation and to reduce the abundance of SCFA-producing bacteria, suggesting that *α*-adrenergic blockers may synergistically improve SCFA levels in patients with coexisting STC and hypertension (Verhaar et al., 2020; Wu et al., 2021). Conversely, microbiota-derived SCFAs exert systemic anti-inflammatory effects that may mitigate hypertension’s impact on gut motility. For example, butyrate and propionate supplementation in experimental models suppresses pro-inflammatory cytokines (TNF-α, IL-6), improves endothelial function, and reduces sympathetic overactivity (Wu et al., 2021). These actions suggest that restoring SCFA signaling could partly alleviate hypertension-related intestinal dysmotility. Psychological stress alters gut microbiota composition within 24–48 h, reducing *Lactobacillus* and *Bifidobacterium* while increasing inflammatory *Enterobacteriaceae*, effects that persist beyond stressor resolution and may explain stress-related constipation (Gareau et al., 2011). Sleep disturbances show a U-shaped relationship with constipation, with both short (<6.5 h) and long (>8.5 h) sleep duration associated with a three-fold increased constipation risk, potentially mediated through circadian disruption of SCFA production and receptor expression (Ohkuma et al., 2024).

## 5 Conclusion and prospects

### 5.1 Summary of the “microbiota–SCFA–neural–immune” integrated mechanism

The microbiota–SCFA–neural–immune axis represents a fundamental regulatory system governing intestinal motility, with dysfunction at any level contributing to STC pathogenesis. This integrated mechanism operates through multiple interconnected pathways: (1) dysbiosis with reduced SCFA-producing bacteria leads to decreased luminal and systemic SCFA levels; (2) SCFA deficiency impairs enterochromaffin cell 5-HT production, disrupts enteric neural signaling, and compromises ICC function; (3) reduced SCFA signaling through FFAR2/FFAR3 diminishes anti-inflammatory responses while promoting barrier dysfunction; (4) the resulting low-grade inflammation further suppresses beneficial bacteria and SCFA production, creating a self-perpetuating cycle (Mansuy-Aubert and Ravussin, 2023). This unified model explains the heterogeneous nature of STC and provides multiple intervention points for therapeutic targeting (Zhang et al., 2022).

### 5.2 Clinical translation potential and precision treatment prospects

#### 5.2.1 SCFA deficiency as the central link between microbiota dysbiosis and motility impairment

The microbiota–SCFA–neural–immune axis constitutes a core regulatory network underlying intestinal motility. Among its components, SCFA deficiency has emerged as the pivotal link connecting dysbiotic microbiota to impaired gut function. Specifically, reduced abundance of SCFA-producing bacteria leads to decreased luminal and systemic SCFA concentrations, which in turn disrupt serotonin synthesis by enterochromaffin cells, impairs enteric neural transmission, and compromises ICC integrity. Additionally, diminished signaling through FFAR2/FFAR3 weakens anti-inflammatory responses and promotes intestinal barrier dysfunction, triggering a self-reinforcing cycle of low-grade inflammation and further SCFA suppression (Zhu et al., 2016) This mechanistic model accounts for the heterogeneity observed in STC and highlights SCFA as a key therapeutic entry point for mechanism-based interventions (Mansuy-Aubert and Ravussin, 2023).

#### 5.2.2 Multi-targeted interventions should integrate microbiota signatures and host genotypes

Translation of microbiota–SCFA research into clinical practice is accelerating. Biomarker-based strategies, such as fecal SCFA profiling, already demonstrate high diagnostic utility for STC (85–90% sensitivity and specificity) (Chen et al., 2024). Point-of-care microbial assays identifying key SCFA-producer deficits can guide rational selection of probiotics, dietary interventions, or FMT, improving response rates by ~40–50% over empirical therapy (Tian et al., 2020). Moreover, combining fecal metabolomics (reflecting SCFA production capacity) with host FFAR2/FFAR3 genotyping enables prediction of response to receptor-targeted drugs with up to 78% accuracy (Bliss et al., 2011). Machine-learning models that incorporate microbiome composition, SCFA profiles, clinical variables, and diet have achieved ~82–90% accuracy in predicting individual treatment outcomes, paving the way for evidence-based, personalized therapy (Han et al., 2024). These advances underscore the necessity of aligning microbiota-targeted interventions with each patient’s unique microbial and genetic context to optimize efficacy.

#### 5.2.3 Future breakthroughs will be driven by multi-omics-guided subtype-specific therapies

To advance personalized treatment for STC, it will be crucial to build comprehensive “microbiota-metabolite-genome” databases. Such resources can power machine learning to stratify patients into endophenotypes – for example, microbiota-responsive, metabolically deficient, or receptor-signal resistant subtypes. Prospective trials can then validate tailored interventions for each subtype (e.g., prioritizing FMT for microbiota-responsive cases or SCFA analogues for metabolically deficient cases). In parallel, we identify key research priorities: (a) standardizing SCFA and microbiome assays to enable data harmonization; (b) developing international biobanks linking microbiota profiles with clinical outcomes; (c) utilizing humanized mouse models colonized with STC patient microbiota for preclinical testing; (d) conducting multicenter RCTs of combination therapies targeting multiple points of the microbiota–SCFA–motility axis; and (e) overcoming translational barriers to bring microbiome diagnostics and therapeutics into routine care (Lucas López et al., 2017). Interdisciplinary collaboration among gastroenterologists, microbiologists, bioinformaticians, and other specialists will be essential to realize the full potential of precision microbiome-based medicine in STC and related disorders (Morrison and Preston, 2016). This integrative review emphasizes a paradigm shift from symptom-based to mechanism-based management of STC. As our understanding of inter-individual variation in microbial ecology and SCFA signaling advances, precision-targeted, subtype-specific therapies will become increasingly viable, offering hope for improved outcomes in this challenging condition.
